# Flow diverters in the treatment of unruptured vertebral artery dissecting aneurysm: A single-center experience

**DOI:** 10.3389/fneur.2023.1050619

**Published:** 2023-02-22

**Authors:** Xiaoyang Lu, Yuansheng Zhang, Hu Zhou, Lipeng Jian, Shi Yin, Tao Li, Wei Huang

**Affiliations:** ^1^Department of Neurosurgery, The First People's Hospital of Yunnan Province, The Affiliated Hospital of Kunming University of Science and Technology, Kunming, Yunnan, China; ^2^The Affiliated Hospital of Kunming University of Science and Technology, Kunming, Yunnan, China

**Keywords:** aneurysm, vertebral artery dissection, flow diverter, endovascular treatment, embolization

## Abstract

**Objective:**

To evaluate the efficacy and safety of flow diverters (FD) in the treatment of vertebral artery dissecting aneurysm (VADA).

**Methods:**

A total of 16 patients with 17 unruptured VADAs treated with FD from January 2017 to May 2021 were included. Data of clinical outcomes and radiographic examination were collected and assessed by the modified Rankin Scale (mRS) and O'Kelly-Marotta (OKM) grading scale.

**Results:**

All patients were treated with a single FD. No perioperative complications occurred. The mean age was 55.1 years old. The mean size of the aneurysm was 10.4 mm. All patients had a favorable occlusion (OKM D + C3) result and the complete occlusion rate in the 6th month was 66.7% (OKM D). The mean clinical follow-up time was 7.8 months, and all patients had a good clinical outcome (mRS = 0). No procedure-related complication occurred at the last follow-up time.

**Conclusion:**

FD is an effective and safe tool for treating unruptured VADA. Long-term prospective studies with a large sample are still needed to confirm these findings in the future.

## Introduction

Vertebral artery dissecting aneurysm (VADA) is a rare and special vascular disease characterized by a dilation of the wall of an artery resulting from tears in the intima and elastic lamina (1). Although VADA is not one of the most commonly encountered intracranial aneurysms, it can cause high mortality and morbidity if ruptures (2). VADA is located in the posterior cranial fossa with complex anatomical structures around it (3). Traditional clipping surgery for treating VADA was reported to have a high complication, morbidity, and mortality rate (4, 5), therefore endovascular treatment (EVT), including simple coiling, stent-assisted coil embolization, or flow diverter (FD) has become the main clinical treatment for VADA in recent years (6). Among these EVT options, FD is a new treatment device based on the hemodynamic mechanism of aneurysm healing (7). It can promote aneurysm healing by improving the local hemodynamics of aneurysms without the need for intra-aneurysm coil embolization (8, 9). In addition, FD greatly simplifies the surgical procedure and reduces the compression effect caused by the aneurysm (10). FD was first approved by the Food and Drug Administration (FDA) for treating large and giant unruptured aneurysms of the internal carotid artery. In view of its excellent efficacy in the treatment of aneurysms, the clinical indication of FD has become more and more widespread and expanded to posterior circulation aneurysms, distal aneurysms, ruptured aneurysms, traumatic aneurysms, and other off-label aneurysms utilizations (11). However, the data regarding FD treating VADA is still controversial and limited so far. The goal of our study is to determine whether FD can be used effectively and safely to treat intracranial unruptured VADA.

## Methods

### Population data

A total of 16 patients with unruptured intracranial VADA from January 2017 to May 2021 in our institution were retrospectively included and reviewed. Digital subtractive angiography (DSA), magnetic resonance (MR), and computerized tomography (CT) angiography are usually used to aid in the diagnosis of VADA. The electronic medical record system provided the data on the patients. The Institutional Review Board of Yunnan First People's Hospital approved this study. Patients' therapeutic decisions (FD, stent-assisted coil embolization, or surgical clipping) were made after considering treatment risks, benefits, and the condition of patients. Patient informed consent is required from every patient before the procedure.

### Postoperative medication

Before the FD deployment procedure, all patients received a 5-day pre-treatment of 75 mg of clopidogrel and 300 mg of aspirin daily as part of dual antiplatelet therapy (DAPT). The response to clopidogrel was monitored *via* P2Y12. Resistance to clopidogrel was defined as inhibition of more than 30% of platelets' P2Y12 receptors. Ticagrelor (90 mg, twice a day) was selected as an alternative to clopidogrel when patients have clopidogrel resistance. A total of 3,000 IU of heparin was administered before the femoral arterial sheath was placed, and then 1,000 IU per hour thereafter. The activated clotting time was monitored throughout the procedure. In the first 6 months after the procedure, the dose of 100 mg of aspirin and 75 mg of clopidogrel were continued daily to use for the first 6 months, after which 100 mg of aspirin was administered for a long time.

### Endovascular procedure protocol

In general, endotracheal intubation anesthesia, a puncture was performed on the common femoral artery on the right side using the Seldinger technique. The right subclavian was usually used for the right-sided VADA, whereas the left subclavian is selected for the left-sided VADA. The subclavian artery was then inserted with a 7Fr shuttle sheath (Cordis, USA). The Pipeline flow diverter (PED^TM^, Medtronic, Dublin, Ireland) or Tubridge flow diverter (TUB^TM^; MicroPort, Shanghai, China) was deployed along the vertebral artery to treat the VADA. In our experience, coiling-assisted FD deployment was conducted only when the aneurysm was acutely ruptured or the maximal aneurysm length was larger than 20 mm (12). As all VADAs in our study are unruptured, no coiling was used during all operations. On a control angiogram, the wall apposition status was assessed, and ultra-compliant balloon or micro guidewire-loop technology was used in the event that better wall apposition was required.

### Patient follow-up

Data on clinical outcomes were collected at the timepoint of admission and last follow-up time and were evaluated with the modified Rankin Scale (mRS) (13). DSA was routinely used for postoperative and 6-month follow-up radiologic evaluation using the O'Kelly Marotta (OKM) grading scale (14) (A—complete filling; B—incomplete filling; C—neck remnant; or D—no filling). The results of DSA were evaluated by two experienced neurointerventional surgeons. Then an annual imaging examination of DSA and computed tomography angiography (CTA) were suggested to be performed for the patients.

## Results

### Baseline patient characteristics

A total number of 16 patients with 17 unruptured VADAs treated with single FD were included in our study cohort. The number of male patients was 10 and the number of female patients was 6. The mean age of all patients was 55.1 years old (ranging from 38 to 74). The mean size of the aneurysm was 10.4 mm (ranging from 4.2 to 16.2). Six Patients (37.5%) were treated with a Pipeline embolization device (PED) and the other 10 patients (62.5%) were operated on using a Tubridge (TUB). Neuropathic symptoms presented in 11 patients, including headache (*n* = 6), vertigo (*n* = 3), neck pain (*n* = 1) and ataxia (*n* = 1), whereas 5 patients were asymptomatic. The mRS of all patients prior to the procedure was zero. Nine of 17 aneurysms were located in the right V4 segment of the vertebral artery (VA), and the other 8 aneurysms were located in the left V4 segment of the VA. Based on the position of the aneurysm and the PICA, three types of VADA were identified: proximal to the PICA, involving the PICA, and distal to the PICA (15). The data on basic patient characteristics were shown in Table 1.

**Table 1:** Clinical features of 16 patients with unruptured vertebral artery dissecting aneurysms and radiologic and clinical follow-up outcomes after flow diversion deployment.

**Patient no**.	**Gender**	**Symptoms**	**Age**	**Aneurysm location**	**Maximal length of the aneurysm (mm)**	**Relation with PICA**	**FD type**	**Clinical FU months**	**Immediate angiography (OKM grade)**	**6-month FU angiography (OKM grade)**	**Last FU mRS**	**Procedure related complication**
1	Male	Headache	41	R V4	8.2	Involving PICA	PED	7	A3	D	0	–
2	Female	Asymptomatic	56	R V4	11.3	Proximal to PICA	TUB	6	B3	D	0	–
3	Male	Vertigo	62	R V4	16.2	Distal to PICA	TUB	8	B3	C3	0	–
4	Male	Headache	43	L V4	15.1	Proximal to PICA	TUB	3	A3	/	0	–
5	Male	Headache	67	R V4	9	Involving PICA	PED	10	A3	D	0	–
6	Female	Asymptomatic	38	L V4	14	Proximal to PICA	TUB	6	C3	D	0	–
7	Male	Headache	48	L V4	8.5	Distal to PICA	TUB	7	B3	D	0	–
8	Female	Asymptomatic	51	L V4	14.6	Proximal to PICA	PED	12	C3	D	0	–
9	Male	Ataxia	55	R V4	7.4	Involving PICA	TUB	8	B3	D	0	–
10	Male	Asymptomatic	70	L V4	13	Distal to PICA	PED	7	B3	C3	0	–
11	Male	Vertigo	52	R V4	8.1	Distal to PICA	TUB	11	B3	C3	0	–
12	Female	Vertigo	62	R V4	6.6, 4.2	Proximal to PICA	TUB	8	A3	D	0	–
13	Female	Headache	74	L V4	11.8	Proximal to PICA	PED	7	A3	C3	0	–
14	Male	Neck pain	54	L V4	9.2	Distal to PICA	TUB	8	A3	D	0	–
15	Female	Headache	61	R V4	7.8	Proximal to PICA	PED	7	A3	C3	0	–
16	Male	Asymptomatic	48	L V4	12.2	Distal to PICA	TUB	9	B3	D	0	–

R, right; L, left; PED, pipeline embolization device; TUB, Tubridge; PICA, posterior inferior cerebellar artery; FU, follow–up; OKM, O'Kelly Marotta; mRS, modified Rankin Scale; “/”, means the follow-up time is <6 months; “–”, means there are no procedure-related complications in the patients.

### Clinical and radiological follow-up outcomes

**Figure 1:**
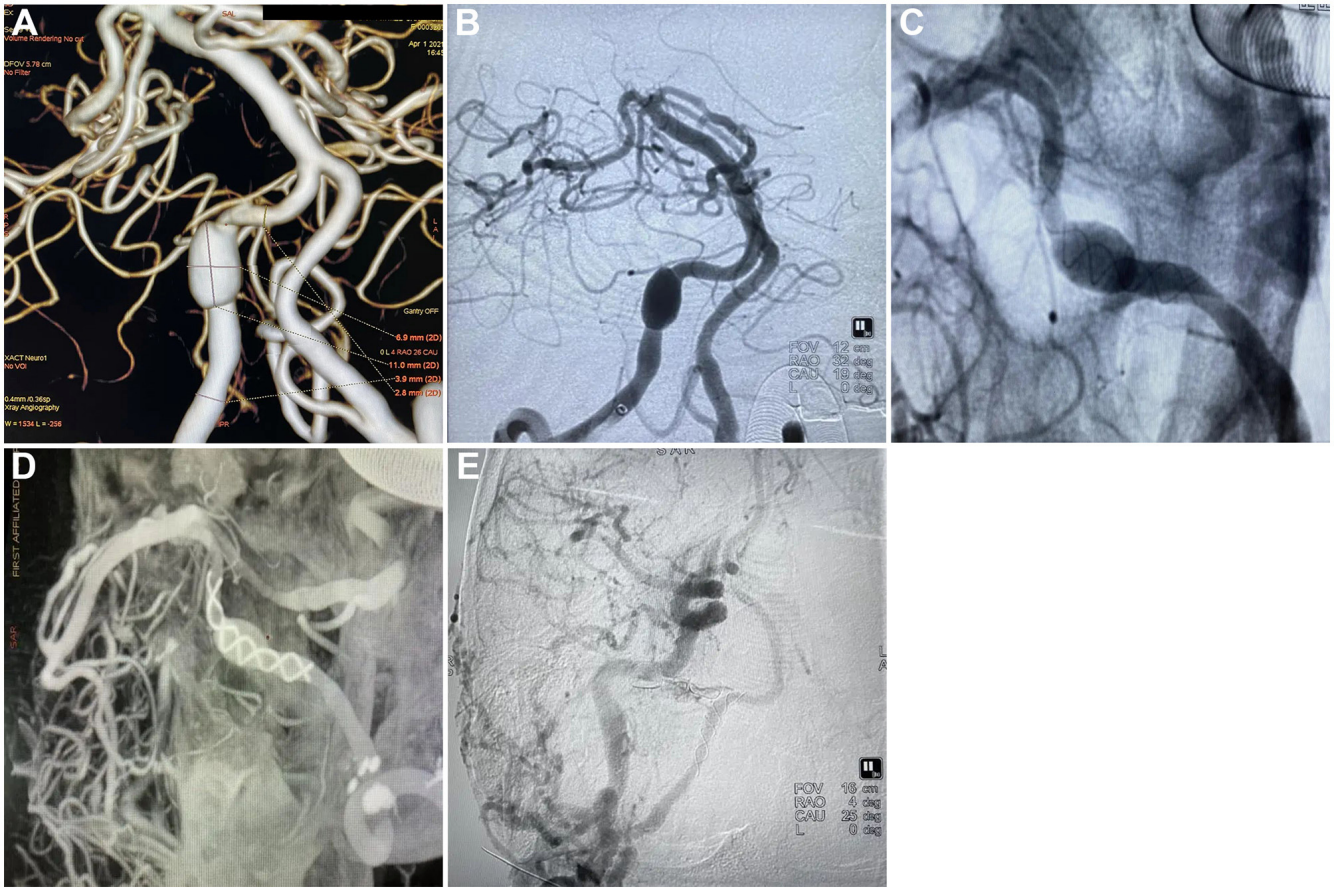
Tubridge treatment on vertebral artery dissecting aneurysm and the follow-up. **(A)** CTA scan showing the dissecting aneurysm in the right vertebral artery, section 4 (V4); **(B)** DSA showing the dissecting aneurysm in the right vertebral artery, section 4 (V4); **(C)** Release of Tubridge flow diverter (3.5 × 55 mm); **(D)** DSA showing a goof wall apposition after final deployment of the flow diverter; **(E)** Follow-up 6 month after the treatment found no silhouette of the dissecting aneurysm.

All 16 patients received a single FD treatment (6 PED and 10 TUB) without additional coils (Figure 1). The operative successful rate was 100%. The results of OKM grade after immediately FD deployment are as follows: A3 (*n* = 6), B3 (*n* = 8), and C3 (*n* = 2), and no perioperative ischemic or hemorrhagic complications happened. DSA follow-up was carried out in 15 patients (93.8%) over 6 months. There was no occlusion of the PICA on the follow-up DSA examination. All patients had a favorable occlusion (OKM grade D + C3) outcome. The complete occlusion rate at 6 months was 66.7% (OKM D, 10/15); the complete occlusion rate of VADA incorporated PICA was 100% (3/3, D); the complete occlusion rate of the aneurysm proximal to PICA or distal to PICA was 66.7% (4/6, D) and 50% (3/6, D), respectively. The mean clinical follow-up time was 7.8 months (3–12 months). A good clinical outcome was achieved for all patients (mRS = 0). All patients were free of procedure-related complications at the last clinical follow-up.

## Discussion

VADA is a rare and treatment-challenging subtype of posterior circulation aneurysms. At present stent-assisted coiling or coil-alone embolization of the parent artery has been considered the main and effective therapy for VADA (16). However, this method also has the risk of aneurysm recurrence and postoperative ischemic complications (17). Before the operation, it is necessary to strictly evaluate whether the diseased side vertebral artery works dominantly for blood supply; whether the contralateral vertebral artery has sufficient blood compensation, and whether the important branch artery such as the posterior inferior cerebellar artery (PICA) is involved (18–20). FD is a new type of endovascular aneurysm treatment device developed in recent years. By reducing the blood flow in the aneurysm, creating an internal-aneurysm thrombus, the luminal healing by epithelialization along the FD stent, and diverting antegrade flow at the lesion artery, it is capable of healing aneurysms clinically (21–23). Many high-quality studies have proven the efficiency and safety of FD in intracranial complicated aneurysm therapy. A pooled analysis of three large studies-ASPIRe (Aneurysm Study of Pipeline in an Observational Registry), PUFS (Pipeline for Uncoilable or Failed Aneurysms Study), and IntrePED (International Retrospective Study of the Pipeline Embolization Device) including 1,092 patients with 1,221 aneurysms concluded that the complete occlusion rates were 75.0%, with a 5.7% major neurological morbidity rate and a 3.3% neurological mortality rate, respectively (24). Similarly, a prospective cohort study focusing on the long-term effectiveness of FD in treating large and giant wide-neck aneurysms found that the long complete occlusion rate was 93.4% at the point of 3-year follow-up, and there were no hemorrhagic or ischemic cerebrovascular events or neurological deaths reported late in the period (25).

However, few studies are reporting the application of FD in VADA treatment by now, and most of them were small sample studies. Oh et al. conducted a retrospective study including 26 VADA patients treated with FD. They found the overall complete occlusion rate was 55.6%, and only two patients occurred with delayed ischemic complications (22). Another retrospective study containing 12 cases with large VADA (>10 mm) showed a favorable occlusion (OKM grade C3 + D) in all 10 patients who were followed up (26). Similar to the previous study, our study found a complete occlusion rate of 66.6% (OKM D) at the sixth-month follow-up. However, further complete occlusion rate in a long follow-up time still needs to be investigated.

A meta-analysis including 15 articles using FD treating posterior circulation non-saccular aneurysms suggested that the periprocedural complications rate was 18% (27). Another comprehensive meta-analysis including 129 cases evaluating the treatment outcome of FD in posterior circulation non-saccular aneurysms reported similar results. They found that 23% of patients suffered periprocedural strokes, and overall mortality and morbidity were 21% and 26%, respectively (28). The patients in our study have no periprocedural stroke complications, but this result must be tested more carefully in studies with larger samples. Although aneurysms in the vertebral artery showed better neurologic outcomes than in other locations (28), periprocedural strokes are still a remarkable risk, and the complications of using FD for VADA treatment still deserve strong alerts. It is important to pay attention to the effect of FD on the blood flow of branch vessels after it has been implanted since it contains a high metal coverage rate, and ischemic events caused by FD-covered branches play an important role in the treatment outcomes (26). It is acknowledged by most clinicians that flow diverter may cause blood stagnation or obstruction of penetrating arteries. The PICA is an important branch of the vertebral artery that may be affected by FD treatment for VADA, causing a fatal cerebellar and brainstem infarction (29). In our study cohort, the patency of PICAs was not influenced in all patients after a 6-months radiologic follow-up.

## Limitations

Our study is a retrospective study with a small patient sample, therefore the statistical analysis was not able to be conducted. More randomized controlled trials or cohort studies with large samples should be conducted to confirm our results. In addition, the follow-up time of the patients in our study is relatively short, a longer follow-up (≥18 months) to evaluate the effect and safety of FD in treating VADA is still necessary for the future.

## Conclusion

FD may be an effective and safe endovascular choice for unruptured VADA treatment as proven by the good clinical outcome and radiological review after 6-month of follow-up. It is important, however, that further long-term and large cohort studies are necessary to confirm these findings.

## Data availability statement

The original contributions presented in the study are included in the article/supplementary material, further inquiries can be directed to the corresponding authors.

## Ethics statement

Ethical review and approval was not required for the study on human participants in accordance with the local legislation and institutional requirements. Written informed consent from the patients/participants or patients/participants legal guardian/next of kin was not required to participate in this study in accordance with the national legislation and the institutional requirements.

## Author contributions

XL, YZ, and HZ collected the data and wrote the manuscript. LJ and SY helped to analyze the data. TL and WH checked the results and coordinated and supervised the study.
